# Enhanced Anti-Herpetic Activity of Valacyclovir Loaded in Sulfobutyl-ether-β-cyclodextrin-decorated Chitosan Nanodroplets

**DOI:** 10.3390/microorganisms11102460

**Published:** 2023-09-30

**Authors:** Monica Argenziano, Irene Arduino, Massimo Rittà, Chiara Molinar, Elisa Feyles, David Lembo, Roberta Cavalli, Manuela Donalisio

**Affiliations:** 1 Department of Drug Science and Technology, University of Turin, Via P. Giuria 9, 10100 Torino, Italy; monica.argenziano@unito.it (M.A.); chiara.molinar@unito.it (C.M.); roberta.cavalli@unito.it (R.C.); 2 Laboratory of Molecular Virology and Antiviral Research, Department of Clinical and Biological Sciences, University of Turin, Regione Gonzole 10, 10043 Orbassano, Italy; massimo.ritta@unito.it (M.R.); elisa.feyles@unito.it (E.F.); manuela.donalisio@unito.it (M.D.)

**Keywords:** valacyclovir, chitosan nanodroplets, sulfobutyl ether-β-cyclodextrin, HSV-2 infection

## Abstract

Valacyclovir (VACV) was developed as a prodrug of the most common anti-herpetic drug Acyclovir (ACV), aiming to enhance its bioavailability. Nevertheless, prolonged VACV oral treatment may lead to the development of important side effects. Nanotechnology-based formulations for vaginal administration represent a promising approach to increase the concentration of the drug at the site of infection, limiting systemic drug exposure and reducing systemic toxicity. In this study, VACV-loaded nanodroplet (ND) formulations, optimized for vaginal delivery, were designed. Cell-based assays were then carried out to evaluate the antiviral activity of VACV loaded in the ND system. The chitosan-shelled ND exhibited an average diameter of about 400 nm and a VACV encapsulation efficiency of approximately 91% and was characterized by a prolonged and sustained release of VACV. Moreover, a modification of chitosan shell with an anionic cyclodextrin, sulfobutyl ether β-cyclodextrin (SBEβCD), as a physical cross-linker, increased the stability and mucoadhesion capability of the nanosystem. Biological experiments showed that SBEβCD-chitosan NDs enhanced VACV antiviral activity against the herpes simplex viruses type 1 and 2, most likely due to the long-term controlled release of VACV loaded in the ND and an improved delivery of the drug in sub-cellular compartments.

## 1. Introduction

Genital herpes is a common sexually transmitted infection characterized by painful genital blisters or ulcers, with an estimated 491 million (13%) people aged 15–49 years affected worldwide [1,2,3,4]. It is mainly caused by herpes simplex virus type 2 (HSV-2) and more rarely by HSV type 1 (HSV-1). A relevant feature of these pathogens is the establishment a lifelong latent infection in neuronal cells that innervate the infected epithelium and periodical reactivation following various environmental cues. Genital herpes can cause severe or fatal infection in newborns through vertical congenital transmission from the mother to the infant [5,6] and can lead to severe complications in immunocompromised patients [7,8,9]. Of note, HSV-2 is strongly correlated to HIV infections [10,11]. In particular, an established HSV-2 genital infection increases the risk of acquiring HIV by approximately three-fold; HIV spread is increased in people with HIV and HSV-2 co-infection, and HSV-2 infection is one of the most common infections in people living with HIV [12,13,14].

The most commonly used anti-herpetic drug is the synthetic nucleoside analogue acyclovir (ACV), usually administered systemically to treat acute infections, which decreases the severity and the frequency of symptoms, although it is not able to eradicate the infection [15,16]. However, due to its short half-life in the bloodstream and incomplete absorption, it must be taken repeatedly throughout the day to be effective. To overcome its poor pharmacokinetic features and thus limiting the dosage, some derivatives of acyclovir have been developed, such as the L-valyl ester of acyclovir, i.e., valacyclovir (VACV), endowed with enhanced bioavailability after oral administration [17]. However, the dose of the drug can reach 1 g/day, resulting in increased unwanted side effects. According to the Biopharmaceutics Classification System (BCS), for VACV (class III drug), permeability is the rate limiting factor for oral absorption [18]. In this context, vaginal administration can represent an approach for improving the local concentration of the drug at the site of infection, limiting systemic drug exposure and consequently reducing undesirable effects [19,20,21].

Nanotechnology-based formulations attracted attention as a promising strategy to improve the local effectiveness of antiviral drugs, increasing drug solubility, protecting the drug from degradation, overcoming the biological barriers and controlling and sustaining the drug release at the site of infection [22,23,24,25]. In addition, nanosystems can efficiently prolong drug retention in the vagina exploiting the high surface area and bioadhesive and mucopenetrating properties [26,27,28,29]. Different polymers have been investigated to design mucoadhesive nanoparticles for vaginal delivery, such as chitosan, a natural polysaccharide with favorable characteristics such biocompatibility, biodegradability, low-toxicity and non-immunogenicity [30,31,32,33].

In this context, chitosan-shelled nanodroplets (ND) have been herein optimized for the vaginal delivery of VACV with the aim of prolonging the retention time, sustaining the release and improving the antiviral effect of the drug. Chitosan ND are spherical nanovesicles composed of a liquid or vaporizable core stabilized by a polysaccharide shell [34]. They have been investigated by different authors as innovative nanoplatforms for the delivery of drug, gases and genetic material [35,36,37]. Previously, our group developed several chitosan ND formulations for the treatment of infectious diseases [38,39,40].

In this study, the chitosan shell of the ND was optimized to increase the stability and the mucoadhesion capability of the nanosystem, using an anionic cyclodextrin, sulfobutyl ether β-cyclodextrin (SBEβCD), as a physical cross-linker. Herein, we report the formulation and the in vitro characterization of the VACV-loaded ND formulations. Biological assays were then carried out to evaluate the improved anti-herpetic activity of VACV delivered through the nanoformulations.

## 2. Materials and Methods

### 2.1. Materials

All reagents employed used were of analytical grade and purchased from Sigma-Aldrich (St. Louis, MO, USA) unless otherwise stated. Soybean lecithin (Epikuron 200^®^) was supplied by Cargill (Hamburg, Germany). Sulfobutyl ether-β-cyclodextrin (Captisol^®^, average molecular weight 2163 Da, average degree of substitution 6.5) was a kind gift from Ligand (San Diego, CA, USA). Chitosan low-molecular weight (degree of deacetylation 75–85%, 50–190 KDa, viscosity: 20–300 cps, 1% solution in 1% *v*/*v* acetic acid) was used.

Methylcellulose, crystal violet, sodium dodecyl-sulfate (SDS), NP-40, sodium deoxycholate, a cocktail of protease inhibitors, Tween 20, glycine, and Triton X-100 were purchased from Sigma-Aldrich. The anti-HSV-1/2 gD monoclonal antibody (clone 2C10—HA025) was purchased from Virusys Corporation (Taneytown, MD, USA). The anti-actin antibody was from Millipore (Burlington, MA, USA) (clone C4—MAB1501R). The antibodies peroxidase-conjugated AffiniPure F(ab’)2 Fragment goat anti-mouse IgG (H+L) and goat anti-rabbit IgG (H+L) were obtained from Jackson ImmunoResearch Laboratories Inc. (West Grove, PA, USA).

### 2.2. Preparation of Nanodroplet Formulations

Nanodroplet (ND) formulations were prepared by purposely tuning a previously described manufacturing method [40], employing decafluoropentane as ND core component and chitosan low molecular weight for the shell. In brief, an Epikuron 200^®^ and palmitic acid (1% *w*/*v*) ethanol solution was mixed with decafluoropentane and ultrapure water and then homogenized (2 min, 24,000 rpm) using an Ultra-Turrax^®^ homogenizer (IKA, Konigswinter, Germany).

Finally, for the polymer shell deposition, a chitosan solution (2% *w*/*v*, pH 5.0) was added dropwise to the sample kept in magnetic stirring. In order to further stabilize the system, the chitosan shell was then physically cross-linked with sulfobutyl ether-β-cyclodextrin (SBE-β-CD) via the addition of SBE-β-CD aqueous solution (1% *w*/*v*) to the preformed chitosan NDs under magnetic stirring. To produce the VACV-loaded NDs, the valacyclovir was added to the Epikuron200^®^ ethanolic solution and the NDs were then prepared as previously described.

Fluorescent NDs were obtained via the addition of 6-coumarin (0.01% *w*/*v*) as a fluorescent marker to the decafluoropentane during the ND preparation or via the use of fluorescein isothiocyanate-labeled chitosan as a coating agent.

### 2.3. Characterization of Nanodroplet Formulations

The physico-chemical parameters (average diameter, polydispersity index and zeta potential) of all ND formulations, either blank or VACV-loaded or fluorescent, were determined via dynamic light scattering (DLS) with a 90Plus particle size analyzer (Brookhaven Instrument Co., Holtsville, NY, USA). The analyses were carried out on samples diluted with filtered water (1:30 *v*/*v*) at a fixed scattering angle of 90° and at 25 ± 1 °C. For the zeta potential measurement, the sample was placed in the electrophoretic cell and an electric field (approximately 15 V/cm) was applied. All the analyses were performed in triplicate.

The yield of the ND samples was calculated by weighting the freeze-dried samples, according to the following equation: Yield (%) = (Effective yield/Theoretical yield) × 100.

The encapsulation efficiency of the VACV-NDs was determined using a centrifugal filter system (Amicon Ultra-0.5 centrifugal filter device). After the centrifugation of a volume of the ND samples (15000 rpm, 30 min) with a Beckman Coulter Allegra 64R Centrifuge, the concentration of free VACVs in the ultrafiltrate was determined via HPLC analysis. The encapsulation efficiency was calculated as the percentage of VACV incorporated into the NDs from the amount of VACVs initially added, using the formula: [(total amount of drug—amount of free drug)/total amount of drug] × 100.

The loading capacity was evaluated on freeze-dried ND samples. Briefly, a weighted amount of freeze-dried VACV-loaded NDs was dissolved in 5 mL of water and after sonication and centrifugation, the supernatant was analyzed via HPLC. The loading capacity was calculated according to the equation: (amount of VACV in NDs/weight of NDs) × 100.

The ND formulation physical stability was studied over time after storage at 4 °C, monitoring the ND physico-chemical parameters and the VACV concentration in NDs up to 6 months.

Moreover, the stability of NDs was evaluated after their incubation in simulated vaginal fluid at pH 4.6, prepared according to Owen and Katz [41], with the following composition: Sodium chloride 3.510 g/L, Potassium hydroxide 1.400 g/L, Calcium hydroxide 0.222 g/L, Bovine serum albumin 0.018 g/L, Lactic acid 2.000 g/L, Acetic acid 1.000 g/L, Glycerol 0.160 g/L, Urea 0.400 g/L, and Glucose 5.000 g/L [42].

#### 2.3.1. Quantitative Determination of Valacyclovir via HPLC Analysis

A high-performance liquid chromatographic analysis with isocratic elution was performed for the quantitative determination of VACV using a PerkinElmer PUMP 250B, equipped with a Flexar UV/Vis LC spectrophotometer detector (PerkinElmer, Waltham, MA, USA). A reversed-phase Agilent TC C18 column (25 cm × 4.6 mm, 5 μm, Agilent Technologies, Santa Clara, CA, USA) and elution with acetonitrile/0.1% formic acid (10:90 *v*/*v* ratio) at a flow rate of 1 mL/min was used. The VACV was detected at 252 nm. The external standard method was used to calculate the VACV concentration. The calibration curve obtained was linear in the concentration range between 0.10 and 10 μg/mL (r^2^ of 0.999).

#### 2.3.2. In Vitro Release Studies

The in vitro release of VACV from the NDs, either with chitosan shell or SBE-β-CD-decorated chitosan shell, was studied via the dialysis bag technique using a cellulose membrane (Spetra/Por, molecular weight cut-off of 14,000 Da; Spectrum Laboratories, Rancho Dominguez, CA, USA). Phosphate-buffered saline (PBS) at pH 4.6 was used as the receiving phase to simulate vaginal fluid. The VACV-loaded ND sample (3 mL) was placed into a dialysis bag and immersed in 50 mL of magnetically stirred receiving phase kept at 37 °C. At fixed time, 1 mL of the receiving phase was withdrawn and replaced with the same volume of fresh PBS to maintain sink conditions. HPLC analysis was performed to determine the VACV concentration in the receiving phase, as previously described. The results, which represented the mean ± standard deviation (SD) of three independent studies, were expressed as % of VACV released over time.

The results of the in vitro release experiments were analyzed using four mathematical kinetic models: the zero-order kinetic model, the first-order kinetic model, the simplified Higuchi model, and the Korsmeyer–Peppas model. In particular, the zero-order kinetic model was evaluated by plotting the cumulative % of the released drug vs. time. The first-order kinetic model was obtained by plotting the log cumulative % of drug remaining vs. time. The Higuchi model was studied by plotting the cumulative % drug release vs. square root of time, whereas the Korsmeyer–Peppas model was analyzed by plotting the log cumulative % drug release vs. log time. A linear regression fit was applied to each plot to obtain the rate constant and correlation values.

#### 2.3.3. Mucoadhesion Capability of Nanodroplet Formulation

The ND formulation mucoadhesive capability was investigated through an in vitro mucin adhesion assay. The ND in vitro binding to mucin was measured via turbidimetric analysis. Briefly, each ND sample was mixed with the same volume of a mucin solution (1 mg/mL) and incubated for 1 h. Then, after the centrifugation of the sample (15 min at 10,000 rpm), the amount of mucin left in the supernatant was quantified by determining the transmittance at 500 nm with an UV spectrophotometer (Du730 spectrophotometer, Beckman, Coulter, Fullerton, CA, USA). The amount of mucin adhering to NDs was determined by subtracting the free mucin content in the supernatant from the total amount of added mucin. The following formula was used to calculate the mucoadhesion percentage (%): Mucoadhesion (%) = ((mucin adhesive to NDs)/total mucin) × 100.

### 2.4. Biological Assays

#### 2.4.1. Cells and Viruses

African green monkey fibroblastoid cells (Vero, ATCC^®^ CCL-81) were cultured in Dulbecco’s modified Eagles’ medium (DMEM) (Sigma-Aldrich) supplemented with 10% heat-inactivated fetal bovine serum (FBS) (Sigma-Aldrich) and 1% (*v*/*v*) antibiotic–antimycotic solution (Sigma-Aldrich) at 37 °C in a humidified 5% CO_2_ atmosphere.

The strains LV and MS (ATCC^®^ VR-540) of HSV-1 and HSV-2, respectively, were used. A laboratory HSV-2 strain with a phenotypic resistance to Acyclovir (HSV-2 ACV-r) was generated as described in a previous work [43]. Viral stocks were produced and titrated on Vero cells via a standard plaque assay, as reported elsewhere [44]. Viral titers were expressed as plaque-forming unit per ml (PFU/mL).

#### 2.4.2. Cell Viability Assay

To test the cytotoxic effect of the drug formulations, the MTS [3-(4,5-dimethylthiazol2-yl)-5-(3-carboxymethoxy-phenyl)-2-(4-sulfophenyl)-2H-tetrazolium] assay was carried out as described by Donalisio et al. [45]. Briefly, Vero cells were treated with serial concentrations of VACV or formulations ranging from 90 µM to 5.6 µM. Cell viability was assessed at 24 h post-treatment using the CellTiter 96 Proliferation Assay Kit (Promega, Madison, WI, USA) by comparing the absorbances of treated cells with those of untreated cells.

#### 2.4.3. Cytotoxicity Assay

The cytotoxicity of the drug formulations on Vero cells was determined using the lactate dehydrogenase (LDH) cytotoxicity assay (CytoTox 96 Non-Radioactive Cytotoxicity Assay, Promega), according to the manufacturer’s instructions. The same experimental conditions of the cell viability assays were used. The percentages of cytotoxicity were calculated in comparison to untreated cells.

#### 2.4.4. HSV Inhibition Assay

The anti-herpetic activity of the drug formulations was evaluated via a plaque reduction assay. Vero cells were seeded at a 1.0 × 10^5^/well density in 24-well plates. The following day, cells were infected with HSV-1, HSV-2 or HSV-2 ACV-r at a multiplicity of infection (MOI) of 0.001 PFU/cells. After a 2 h incubation at 37 °C, the viral inoculum was removed and the cells were treated with serial dilutions of the drug formulations (from 14.8 µM to 0.23 µM) in DMEM containing 1.2% methylcellulose (Sigma-Aldrich). VACV was tested in parallel as a reference. At 24 h post-infection (hpi) (for HSV-2 and HSV-2 ACV-r) or 48 hpi (for HSV-1), the cells were subjected to fixing and staining with 0.1% crystal violet in 20% ethanol, and the viral plaques were microscopically counted. The inhibition of infectivity by the antiviral treatment was calculated as percentage of viral plaques in the treated wells in comparison to the untreated controls. The size of HSV-2 plaques was determined in at least 10 microscopic fields for each compound concentration (VACV, SBEβCD-ND-VACV and SBEβCD-ND) using an Axiovert 200 inverted microscope (Zeiss, Jena, Germany), Infinity3 microscope camera (Lumenera, Ottawa, ON, Canada) and Infinity Capture software 6.3 (Lumenera). Data were analyzed with Infinity Analyze software 7.0 (Lumenera) and ImageJ software 1.5 (Bethesda, MD, USA).

#### 2.4.5. Post-Infection Kinetics Assay

This assay was performed to evaluate the anti-HSV-2 activity of the SBEβCD-ND-VACV when administered at 1 and 6 h after HSV-2 infection. VACV was tested in parallel as a reference. Vero cells in 24-well plates (1.0 × 10^5^ cells/well) were infected with HSV-2 at MOI 0.001 PFU/cell for 2 h at 37 °C. After viral adsorption, the viral inoculum was removed and cells were overlaid with culture medium. After 1 or 6 h, the serial dilutions of SBEβCD-ND-VACV and VACV were added in 1.2% methylcellulose DMEM and incubated at 37 °C for 24 h, and cells were treated with the virus inhibition assay. The number of viral plaques were microscopically counted, and data were reported as the percentage of infection in comparison to the infected untreated controls.

#### 2.4.6. Virus Yield Reduction Assay

The ability of drug formulations to inhibit the production of HSV-2 progeny was investigated with this experiment. Vero cells (1.0 × 10^5^/well) were infected with HSV-2 at MOI 0.01 PFU/cells at 37 °C for 2 h. Subsequently, the cells were treated with serial dilutions of drug formulations or VACV as a reference. After an extensive cytopathic effect appeared in untreated wells, the cultures were harvested, pooled and clarified via centrifugation. Viral titers were determined through plaque assays and expressed as PFU/mL.

#### 2.4.7. Immunoblotting

The inhibition of viral protein expression was investigated by means of immunoblotting. Vero cells were infected with HSV-2 (MOI of 1 PFU/cell) for 2 h at 37 °C and then treated with a high inhibitory concentration (30 µM) of SBEβCD-ND-VACV and VACV. After 4 h incubation at 37 °C, cells were harvested and incubated in lysis buffer (150 mM NaCl, 50 mM Tris-HCl pH 8, 0.1% SDS, 1% NP-40, 0.5% sodium deoxycholate and inhibitors). Proteins were collected through centrifugation, as described elsewhere [46], and stored until use at ™80 °C. For immunoblotting, protein extracts were separated using SDS-polyacrylamide gel electrophoresis and transferred to Immobilon-P membranes (Millipore) and were incubated overnight with a blocking buffer (5% non-fat dry milk, 10 mM Tris-Cl pH 7.5, 100 mM NaCl, 0.1% Tween 20). Membranes were then blotted with anti-HSV-2 gD glycoprotein and anti-actin mouse monoclonal antibodies and secondary horseradish peroxidase-conjugated antibody. Immunocomplexes were visualized via enhanced chemiluminescence (Super Signal™, Thermo Scientific, Waltham, MA, USA) and the ChemiDoc™ Touch Imaging System (Bio-Rad Laboratories, Inc., Hercules, CA, USA), and analyzed with the Image Lab Software 6.1 (Biorad).

#### 2.4.8. Assessment of Cellular Penetration of NDs via Confocal Laser Microscopy

Vero cells were seeded on the coverslip in 24-well plates (3 × 10^4^ cells/well). The next day, cells were treated with fluorescent (6-coumarin)-labeled SBEβCD-ND-VACV (at 50 µM) for 5 min, 30 min, 1 h, 3 h and 16 h. After treatment, 5 washes with PBS1X were performed, coverslips were mounted and observed on the confocal laser microscope (LSM800; Carl Zeiss, Jena, Germany) and images of green cells were acquired.

#### 2.4.9. Determination of VACV Concentration in Vero Cells

The intracellular content of VACV was evaluated through HPLC analysis in whole cell extracts and in nuclear compartments. To obtain extracts, 60 mm culture dish pre-seeded Vero cells (5 × 10^5^ cells/well) were treated with 10 µM and 50 µM of SBEβCD-ND-VACV or VACV for 1 h, 3 h or 24 h at 37 °C.

To obtain whole cell extracts, the treated cells were washed with PBS1X 3 times, and trypsinized and lysed with a saturated solution of ammonium sulphate at 4 °C. Cellular debris were pelleted via centrifugation at 4 °C for 10 min at 13,000 rpm, and supernatants were collected and stored at −80 °C.

Nuclear extracts were obtained as described in a previous work [47]. Briefly, harvested cells were incubated for 15 min at 4 °C in hypotonic buffer and then disrupted through repeated passages into a syringe and centrifuged at 11,000× *g* for 20 min to obtain nuclear pellets. Nuclei were rinsed once in hypotonic buffer, resuspended in hypertonic buffer, and incubated for 30 min at 4 °C with gentle agitation. After centrifugation at 15,000× *g* for 10 min, supernatants were collected and stored at −80 °C.

Whole-cell extracts or nuclear extracts were thawed and after centrifugation (13,000 rpm, 15 min, 10 °C) and suitable dilution with the mobile phase were analyzed through HPLC, as described above, to determine the concentration of VACV.

#### 2.4.10. Statistical Analysis

All analyses were conducted using GraphPad Prism (Graph-Pad Software 9.0, San Diego, CA, USA). Half-maximal effective concentrations (EC_50_), concentrations that reduced viral infectivity by 90% (EC_90_), and 50% cytotoxic concentrations (CC_50_) with 95% confidence intervals (CI) were calculated via regression analysis. Dose–response curves were compared via F-test as appropriate. One-way analysis of variance (ANOVA) followed by a Bonferroni post hoc test was used to compare plaque size of untreated and treated samples in the HSV inhibition assays and viral titers in the virus yield reduction assays. In post-infection kinetics assays, viral infectivity (%) between VACV- and formulation-treated samples for each concentration was compared using Student’s *t*-test. Significance was set for *p*-values of < 0.05 (*), <0.01 (**) and <0.001 (***). All experiments were performed in triplicate, unless stated otherwise.

## 3. Results and Discussion

### 3.1. Preparation and Characterization of Nanodroplet Formulations

Two series of NDs were obtained for VACV delivery, one comprising only a chitosan shell (ND) and the other a cross-linked chitosan/cyclodextrin shell (SBEβCD-ND). The SBEβCD-ND shell consisted of a low-molecular weight chitosan physically cross-linked with an anionic cyclodextrin such as SBEβCD. SBEβCD is a polyanionic β-cyclodextrin derivative with six to seven sulfobutyl-ether groups per cyclodextrin molecule, having increased aqueous solubility and inclusion capability compared to the parent β-CD. The negatively charged sulfobutyl-ether groups of SBEβCD can electrostatically interact with the cationic amino groups of chitosan present on the ND surface, leading to a more rigid and thick cross-linked shell.

Table 1 reports the physico-chemical characteristics of all the ND formulation evaluated by DLS analysis. All types of NDs had sizes of about 400 nm, good polydispersity indices and positive surface charge. The positive zeta potential values are determined through the cationic amino groups of chitosan chains on the outer layer of the nanostructure.

The presence of the ND chitosan shell was also previously established via TEM analysis, which showed a clear polymer shell on the ND surface [40]. To further confirm the presence of the chitosan shell, fluorescein isothiocyanate-labeled chitosan was prepared and used as coating agent in the ND preparation. A well-defined fluorescent shell was observed in the ND nanostructure via fluorescence microscopy (data not shown). The marked reduction in zeta potential values (about 30%) of SBEβCD-ND compared to ND confirmed the electrostatic interaction between chitosan and SBEβCD (Table 1).

The combination of cationic polysaccharides with polyvalent anions such as SBEβCD has largely been exploited for the preparation of micro–nanoparticles through ionic-crosslinking [48,49,50,51]. A ND system composed of a hybrid chitosan/SBEβCD shell was previously developed by our group for the delivery of acyclovir [38], exploiting the CD complexation capability for the loading of the drug. In this work, the chitosan/SBEβCD shell was designed to achieve a more stable ND system and provide a prolonged VACV release kinetics. The presence of cyclodextrin in the ND shell might also exert a possible antiviral synergic effect with the drug. Moreover, it is worth noting that the electrostatic interactions of cationic polymers with SBEβCD produce mucoadhesive systems able to interact with the vaginal mucus layer forming sustained-release formulations [52].

The mucoadhesion capability of both the ND formulations was investigated via an in vitro mucin adhesion assay and a 92.4 ± 0.5% and 96.8 ± 0.4% of mucoadhesion was obtained for ND and SBEβCD-ND, respectively. The presence of VACV encapsulated in the NDs did not interfere with mucoadhesive properties.

This result indicated the interaction of chitosan in the ND shell with mucin, which mainly involves electrostatic interactions, hydrogen bonds and hydrophobic effects [53,54]. Mucoadhesion is an important feature for vaginal antiviral delivery since it can increase the residence time of loaded nanoparticles at the infection site, leading to an improvement in drug absorption and a greater therapeutic effect [55,56].

The VACV was efficiently loaded in NDs, with a good encapsulation efficiency (90.5 and 91.2% for ND-VACV and SBEβCD-ND-VACV, respectively). The loading capacity was 5.20 and 5.28% for ND-VACV and SBEβCD-ND-VACV, respectively. The yield percentage ranged from 94.05 to 94.35 for ND-VACV and SBEβCD-ND-VACV. The incorporation of VACV in NDs did not significantly modify their physico-chemical features, such as size and zeta potential.

**Figure 1:**
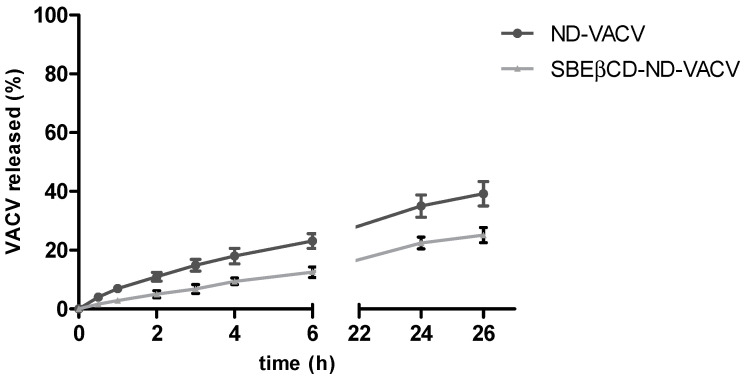
In vitro release kinetics of VACV from VACV-loaded NDs. The results are expressed as the mean ± SD (n = 3).

Figure 1 shows the in vitro release kinetics of VACV from the two ND formulations. The VACV was released from both the NDs with a prolonged release kinetics without initial burst effect, confirming the encapsulation of the drug in ND systems.

The SBEβCD cross-linked shell was able to provide a slower in vitro release profile of VACV than the one of ND, reaching the 12.5 and 22% of VACV released after 6 and 24 h, respectively.

The VACV release profiles from the two types of NDs were fitted to four different kinetic models (zero order, first order, Higuchi and Korsmeyer–Peppas) to assess the mechanism of drug release. For each model, the rate constant and correlation values were obtained by applying a linear regression fit (Table 2).

The Higuchi model showed the highest correlation with the experimental results, having the highest R^2^ value for both the formulations (0.976 and 0.988 for ND-VACV and SBEβCD-ND-VACV, respectively). This result suggested that the release kinetics are controlled by the diffusion through the polymer shell.

A similar behavior was observed for the in vitro release of prednisolone phosphate from a new ND formulation designed by our group, consisting of a hard shell composed of glycol chitosan cross-linked with tripolyphosphate ions [57].

It is of note that the aqueous nanosuspension formulations were stable for up to 6 months stored at 4 °C. Indeed, no significant modification in the ND size and surface charge was observed and the concentration of VACV remained stable in both ND formulations.

Moreover, the physical stability in simulated vaginal fluid was assessed. No aggregation phenomena were observed and no change in ND size was registered, confirming the suitability of NDs as a vaginal drug delivery system.

### 3.2. Assessment of the Anti-Herpetic Activity of ND Formulations

**Figure 2:**
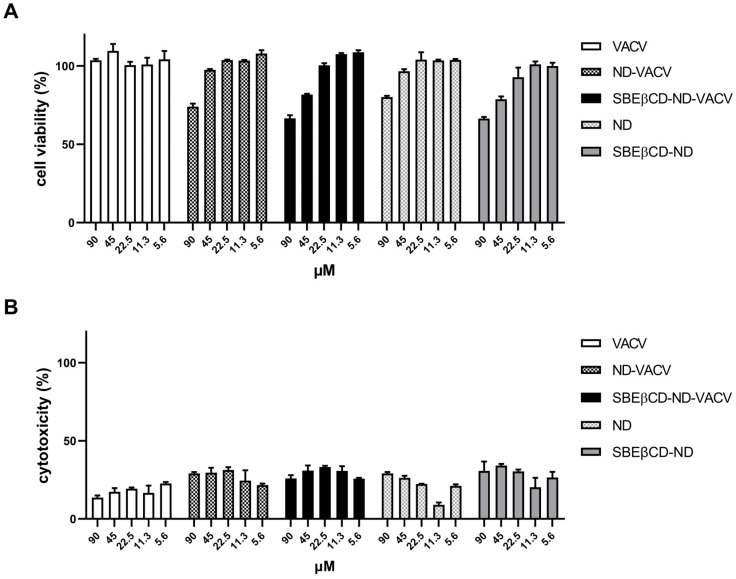
Effect of VACV and ND formulations on uninfected Vero cells. Uninfected Vero cells were incubated with decreasing concentrations of the drug, incubated at 37 °C, and 24 h later, MTS assay (**A**) or LDH assay (**B**) was used to assess cell viability or cytotoxicity, respectively. X-axis: VACV concentration (µM). Y-axis: (**A**) cell viability (% of untreated control); (**B**) cytotoxicity (% of untreated control). Error bars represent SEM in three independent experiments.

Firstly, cell viability and cytotoxicity assays were performed to assess the possible toxic effects of NDs on the cell line used for all biological experiments, i.e., Vero cells. As reported in Figure 2A, results of cell viability assays showed that all compounds tested exhibited 50% cytotoxic concentration (CC_50_) values of >90 µM. A weak reduction in cell proliferation (<80%) was observed only at the highest concentration tested for the ND formulations. As for the cytotoxicity assays, we evaluated the toxic effect of VACV and NDs on a cell monolayer by measuring the release of the cytosolic enzyme lactate dehydrogenase (LDH) into the cell culture supernatant. These assays confirmed the CC_50_s of VACV and NDs (>90 µM) (Figure 2B). Thus, all following cell-based experiments were performed at safe, non-cytotoxic concentrations of the drug in order to exclude whether any inhibitory activity could be due to cellular alterations induced by the drug treatment.

**Figure 3:**
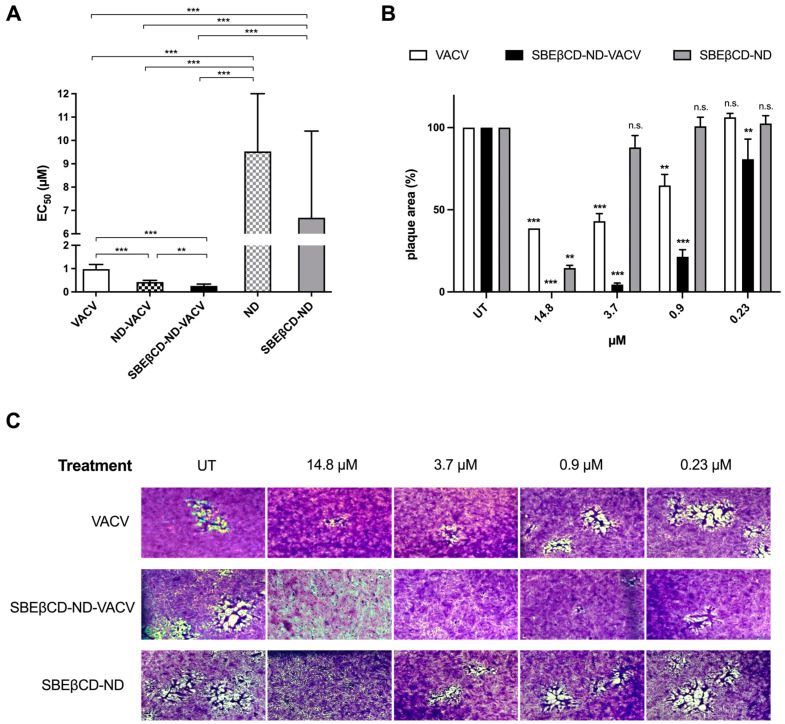
Effect of VACV and ND formulations on HSV-2 infectivity. (**A**) Anti-HSV-2 activity of VACV and ND formulations. Plaque reduction assays were carried out adding the drug serially diluted to cells for 24 h after removal of virus inoculum, as described in the Section 2. Plaque formation was assessed 24 h after infection, and dose–response curves were calculated. Results are reported as half-maximal effective concentrations (EC_50_) and 95% confidence intervals. Data were compared via F-test. ** *p* < 0.01; *** *p* < 0.001. (**B**,**C**) Effect of VACV and SBEβCD-ND formulations on cell-to-cell spread of HSV-2. (**B**) The bar chart shows the percentage of HSV-2 plaque area of treated wells compared to that of untreated control wells as a function of the concentration of drug in the plaque reduction assay. VACV (white)-, SBEβCD-ND-VACV (black)- and SBEβCD-ND (grey)-treated samples were compared at each dose with one-way ANOVA. n.s.: not significant; ** *p* < 0.01; *** *p* < 0.001. (**C**) Representative HSV-2 plaques in Vero cells observed in the plaque reduction assay are reported for untreated control and for the following concentrations of drug: 14.8, 3.7, 0.9, and 0.23 µM. Magnification, 50×. UT: untreated. The pictures and bar chart are representative of ≥10 plaques per condition.

A set of antiviral assays was performed on Vero cells to evaluate the inhibitory activity of the different ND formulations against HSV-2 infection, compared to that of VACV alone. This assay, i.e., a plaque reduction assay, was finalized to quantify the antiviral effect of the drug, evaluating its ability to reduce the number of viral plaques on the cell monolayer, compared to infected untreated control. Briefly, cells were infected for 2 h, then treated with increasing concentrations of VACV or NDs in 1.2% methylcellulose medium and incubated for 24 h at 37 °C to allow HSV-2 replication. The drug was added only after infection as the antiviral action of VACV (i.e., the inhibition of the viral DNA polymerase) is exerted during intracellular viral replication. After incubation, viral plaques were stained and counted in order to generate dose–response curves and determine the EC_50_ values (Figure 3A and Table 3). In accordance with the efficacy of VACV reported in the literature [58], free VACV showed an EC_50_ of 0.98 µM against HSV-2. The ND formulations loaded with VACV (ND-VACV and SBEβCD-ND-VACV) were significantly more active than VACV alone in inhibiting HSV-2 replication (*** *p* < 0.001). Moreover, the antiviral activity of SBEβCD-ND-VACV was higher than that of ND-VACV, with EC_50_s of 0.26 and 0.43 µM, respectively (** *p* < 0.01). Of note, blank NDs were shown to be antivirally active, although to a lesser extent compared to drug-loaded formulations, with EC_50_ values 10-fold higher than VACV-loaded NDs (*** *p* < 0.001) and EC_90_ values not assessable. Hence, we cannot exclude that an antiviral effect of the shell may partially contribute to the activity of the drug-loaded nanodroplets.

Although the modification of the chitosan shell with the anionic cyclodextrin conferred an increase in antiviral potency to the NDs, the SBEβCD solution as such did not exert inhibition against HSV-2 (data not shown). This result is in contrast with previous research that reported a putative antiviral activity of sulfonated cyclodextrins and their derivatives [59,60].

Next, we assessed the spectrum of antiviral activity of NDs against other herpes simplex viruses (Table 3). Specifically, NDs were tested against the closely related HSV-1. Table 3 shows that NDs loaded with VACV exhibited stronger inhibitory effect than VACV alone, similarly to that against HSV-2. Interestingly, blank NDs did not exert antiviral activity against HSV-1, whereas partially active against HSV-2. Indeed, the difference in sensitivity of HSV-1 and HSV-2 to antiviral compounds has already been reported in the literature [46]. This could be ascribed to the specific viral strain or cell line used in the experiments or to different biological functions of some viral proteins, as previously reported [61,62]. Moreover, ND formulations were assayed against an acyclovir-resistant strain of HSV-2 (HSV-2 ACV-r), previously generated in our laboratory [43]. As expected, HSV-2 ACV-r was insensitive to VACV alone. On the contrary, all ND formulations, both loaded with VACV and blank, were partially active against the resistant strain. This result could be due to an intrinsic antiviral effect of the ND, which was also observed against the wildtype HSV-2 strain and could not involve the same viral target of VACV.

### 3.3. Investigation of the Antiviral Activity of the SBEβCD-ND-VACV Formulation against HSV-2

For the following experiments that aimed to characterize more deeply NDs’ antiviral activity, we selected only the SBEβCD-ND-VACV formulation as it exhibited a higher inhibitory effect against HSV-2, compared to the ND formulation without cyclodextrins.

The bright-field optical microscope examination of stained cell monolayers in the plaque reduction assay showed that VACV reduced in a dose-dependent manner not only the viral plaque number but also the viral plaque area (Figure 3B,C). These data indicate that the drug inhibits cell-to-cell spread, most likely due to the blocking of the production of infectious viral progeny by the antiviral drug. Interestingly, the SBEβCD-ND-VACV formulation was more active in inhibiting viral spread than VACV alone. Of note, no viral plaques were detected at the highest tested concentration, i.e., 14.8 μM, for SBEβCD-ND-VACV. At all other tested doses, the viral plaque area was significantly reduced compared to untreated control and to VACV. These observations confirmed the improvement of antiviral activity of the VACV-loaded ND formulation, compared to VACV alone. Of note, the blank formulation, SBEβCD-ND, showed partial activity, with a reduction in plaque size only at the highest tested dose, i.e., 14.8 μM. This result may be related to the presence on the blank ND surface of SBEβCD molecules closely linked to chitosan. Taking into account the strong interactions between the cyclodextrin and chitosan, SBEβCD moieties could be exposed towards the external environment. This decorated shell of SBEβCD-ND can mimic negatively charged polymers carrying conjugated sulfonate groups. Of note, the antiviral activity against HSV-2 of sulfonated cyclodextrin polymers has been previously shown [63].

**Figure 4:**
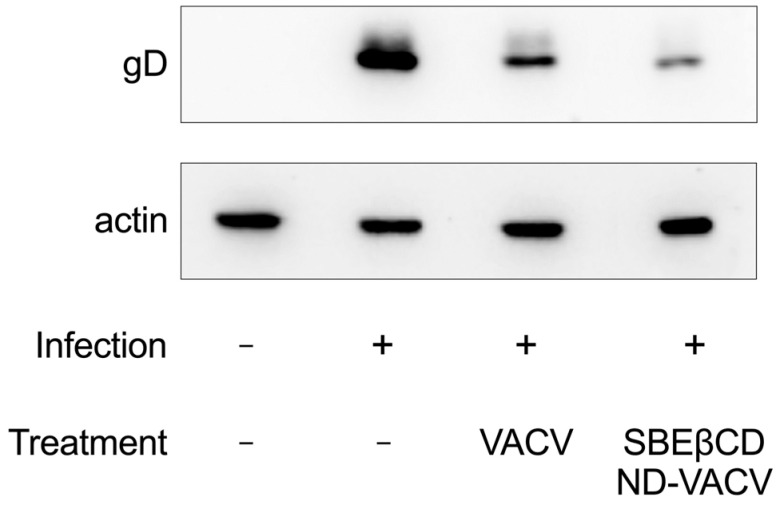
Effect of VACV and SBEβCD-ND-VACV formulation on HSV-2 protein expression. Protein extracts from Vero cells infected with HSV-2 (MOI 1 PFU/cell) and treated with the drug at the fixed dose of 30 µM were obtained 4 h post-infection, and levels of HSV-2 glycoprotein D (gD) were investigated via immunoblotting. Actin was used as the internal control.

Next, the effect of treatment on the expression of viral proteins (i.e., the late glycoprotein D, gD) was investigated via Western blotting. Results are reported in Figure 4. As expected, VACV treatment strongly reduced the expression of gD, as the antiviral prodrug is a known inhibitor of viral replication, which occurs prior to late gene expression. Notably, gD expression was inhibited by theSBEβCD-ND-VACV formulation more than by VACV alone, further confirming the enhanced antiviral activity of the formulation in comparison with VACV alone.

**Figure 5:**
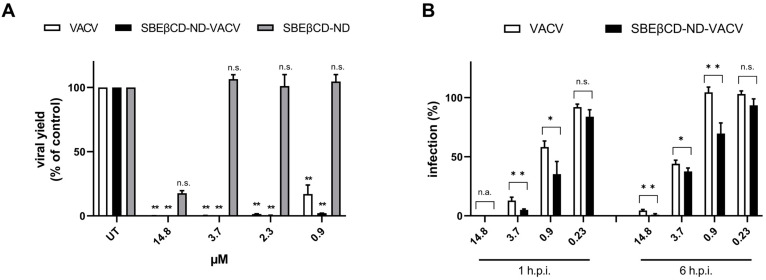
Effect of VACV and SBEβCD-ND-VACV formulation on later steps of the HSV-2 replicative cycle. (**A**) Virus yield reduction assays. Viral progeny was harvested when full cytopathic effect was displayed in untreated control samples, and viral titers were determined via standard plaque assay. Results are reported as percentages of viral titers (PFU/mL) of treated samples in comparison to untreated controls (UT). Data are shown as mean ± SEM and were analyzed via one-way ANOVA. n.s., not significant; ** *p* < 0.01. (**B**) Post-infection kinetic assays. The anti-HSV-2 activity of drug formulations was evaluated when administered at 1 and 6 h after the removal of HSV-2 inoculum. Plaque formation was quantified. Data are shown as mean ± SEM and were compared using Student’s *t*-test. n.s., not significant; * *p* < 0.05; ** *p* < 0.01.

To characterize the activity of the drug formulation over time, we firstly assessed the ability of VACV to reduce the production of HSV-2 infectious progeny using virus yield reduction assay. Figure 5A shows that VACV and SBEβCD-ND-VACV formulation were able to strongly inhibit HSV-2 yield at all effective doses tested (** *p* < 0.01). On the contrary, in this more stringent experimental setting, the blank formulation did not exert antiviral activity, although a partial inhibitory effect was observed in previous experiments. All viral titers are reported in Supplementary Table S1. It is of note that the drug formulation was more active than VACV, as evidenced by the EC_90_ value (0.54 µM for ND and 1.19 µM for VACV) and the higher titer reduction at sub-optimal doses compared to untreated controls (2.5- and 1.9-log reduction at 2.3 µM and 1.7- and 0.8-log reduction at 0.9 µM for ND and VACV, respectively). Next, we investigated whether the ND formulation maintained its improved inhibitory effect when added at late time points after HSV-2 infection, compared to VACV alone. As shown in Figure 5B, SBEβCD-ND-VACV was still effective when added up to 6 h after HSV-2 infection, with EC_50_ values of 0.62 µM and 2.01 µM for 1 and 6 h, respectively, whereas VACV showed a weaker inhibitory activity (1.11 µM and 3.31 µM).

**Figure 6:**
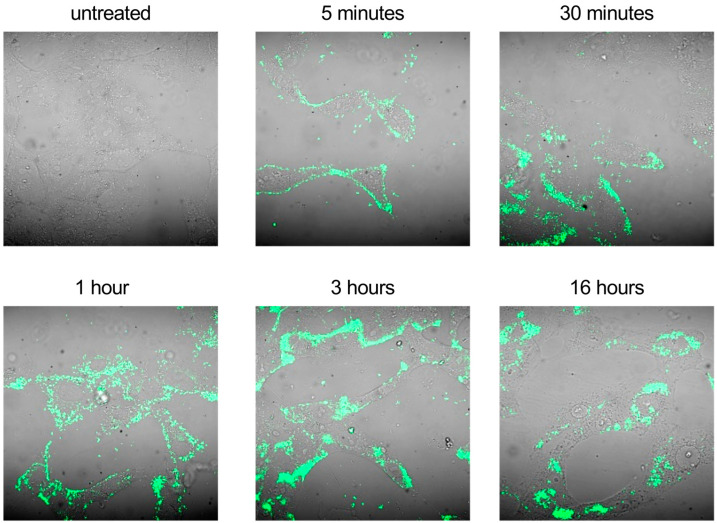
Cell uptake of fluorescent SBEβCD-ND-VACV complex. Uninfected Vero cells were treated with fluorescent SBEβCD-ND-VACV complex at 50 µM for 5 min, 30 min, 1 h, 3 h and 16 h. Fluorescent complex uptake in live not-fixated cells was visualized in green via confocal laser microscopy. Control sample (untreated) was incubated with culture medium alone. Magnification, 400×.

Based on these experimental data, we hypothesized that the higher antiviral activity of the drug formulation could be mainly ascribed to a more efficient intracellular delivery of the drug by the nanodroplets. To investigate this hypothesis, we first confirmed whether SBEβCD-ND-VACV was able to penetrate intracellularly. Confocal microscopic observation of fluorescent ND-treated live cells at different time points (Figure 6) revealed that the drug formulation was able to bind Vero cells as soon as 5 min after treatment and started to enter cells after a 30 min treatment. At 1 h post-treatment, the fluorescent signal appeared localized in the cytoplasm, distributing homogeneously in the cytoplasm after 3 h. Interestingly, 16 h post-treatment cells showed a well-defined perinuclear localization of the fluorescent ND.

**Figure 7:**
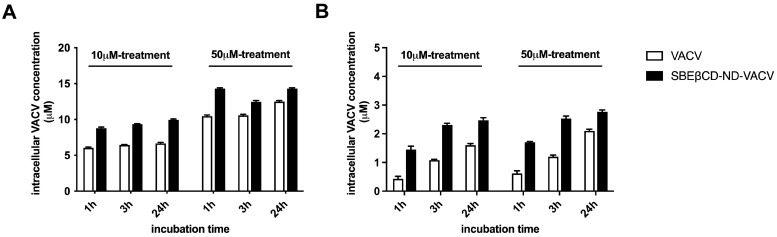
Intracellular VACV concentration (μM) in Vero cells, in whole-cell lysates (**A**) and in nuclear compartment (**B).** Vero cells were treated with 10 μM and 50 μM of SBEβCD-ND-VACV and VACV for 1, 3 and 24 h at 37 °C. Then, the cells were lysed as described in the Section 2 and cell lysates were analyzed using HPLC. The results are represented as mean ± SD.

Next, we investigated the cellular uptake over time of VACV delivered by the ND or administered alone in whole-cell extracts of treated cells at different times post-infection. Figure 7A shows that a higher intracellular concentration of VACV was found in whole-cell extracts after treatment with SBEβCD-ND-VACV compared to VACV alone, at all times and doses tested. In addition, we quantified the VACV concentration in the nuclear compartment of treated cells, since the antiviral activity of VACV is exerted in the nucleus where the viral DNA polymerase acts. As depicted in Figure 7B, treatment with NDs enhanced the nuclear accumulation of VACV, compared to the treatment with the drug alone.

## 4. Conclusions

In this study, decorated SBEβCD-chitosan NDs were obtained for the prolonged and sustained release of VACV. The ND shell was purposely optimized to increase the stability of the system, confer mucoadhesion capability and control the drug release. In vitro cell-based experiments showed that SBEβCD-chitosan NDs enhanced VACV antiviral activity against wildtype herpes simplex viruses type 1 and 2, which might be mainly ascribed to the long-term controlled release of VACV loaded in the ND and to an improved delivery of the drug in sub-cellular compartments. Altogether, this work demonstrates the validity of nanoformulations as a delivery system for topical drug administration and paves the way for developing an effective microbicide for genital herpes infections.

## Figures and Tables

**Table 1:** Physico-chemical parameters of ND formulations.

Formulation	Average Diameter ± SD (nm)	Polydispersity Index ± SD	Zeta Potential ± SD (mV)
ND	395.6 ± 15.4	0.23 ± 0.02	32.10 ± 3.25
SBEβCD-ND	384.8 ± 20.3	0.22 ± 0.01	20.55 ± 2.44
ND-VACV	398.8 ± 12.4	0.21 ± 0.02	30.46 ± 3.01
SBEβCD-ND-VACV	390.4 ± 22.5	0.22 ± 0.02	21.12 ± 2.87
Fluorescent ND	396.5 ± 18.8	0.20 ± 0.03	31.73 ± 3.22
Fluorescent SBEβCD-ND	388.3 ± 20.2	0.23 ± 0.02	22.05 ± 1.96

Results presented as mean ± standard deviation (SD) (n = 3).

**Table 2:** Kinetic models of drug release profiles.

Sample	Regression Coefficient (R^2^)			
	*Zero-order*	*First-order*	*Higuchi model*	*Korsmeyer–Peppas model*
ND-VACV	0.8668	0.9068	0.9757	0.8565
SBEβCD-ND-VACV	0.9321	0.9481	0.9879	0.9783

**Table 3:** Antiviral activity of VACV and ND formulations against HSVs.

Virus	Formulation	EC_50_^a^ (95% CI ^b^)	EC_90_^c^ (95% CI)
HSV-2	VACV	0.98 (0.81–1.18)	6.42 (4.22–9.77)
	ND-VACV	0.43 (0.37–0.50)	2.65 (1.89–3.71)
	SBEβCD-ND-VACV	0.26 (0.20–0.34)	3.19 (1.73–5.88)
	ND	9.53 (6.07–15.0)	n.a.
	SBEβCD-ND	6.69 (4.30–10.4)	n.a.
HSV-1	VACV	0.45 (0.36–0.56)	1.83 (1.22–2.74)
	ND-VACV	0.27 (0.22–0.32)	0.50 (0.20–1.22)
	SBEβCD-ND-VACV	0.27 (0.20–0.36)	0.75 (0.39–1.45)
	ND	n.a.	n.a.
	SBEβCD-ND	n.a.	n.a.
HSV-2 ACV-r	VACV	n.a.	n.a.
	ND-VACV	1.97 (0.88–4.42)	4.23 (1.46–12.2)
	SBEβCD-ND-VACV	1.92 (1.34–2.75)	3.84 (2.43–6.07)
	ND	1.41 (1.02–1.95)	4.48 (2.16–9.30)
	SBEβCD-ND	3.28 (2.44–4.40)	5.59 (2.08–15.0)

n.a., not assessable. ^a^ half maximal effective concentration. ^b^ 95% confidence intervals. ^c^ 90% effective concentration.

## Data Availability

All data generated or analyzed during this study are included in the published article.

## Supplementary Materials

The following supporting information can be downloaded at: https://www.mdpi.com/article/10.3390/microorganisms11102460/s1, Table S1: Viral titers of virus yield reduction assays, expressed as Log10(PFU/mL) ± standard deviation (SD).

## References

[1] AlMukdad S., Harfouche M., Farooqui U.S., Aldos L., Abu-Raddad L.J. (2023). Epidemiology of Herpes Simplex Virus Type 1 and Genital Herpes in Australia and New Zealand: Systematic Review, Meta-Analyses and Meta-Regressions. Epidemiol. Infect..

[2] Harfouche M., Alareeki A., Osman A.M., Alaama A.S., Hermez J.G., Abu-Raddad L.J. (2023). Epidemiology of Herpes Simplex Virus Type 2 in the Middle East and North Africa: Systematic Review, Meta-Analyses, and Meta-Regressions. J. Med. Virol..

[3] Alareeki A., Osman A.M.M., Khandakji M.N., Looker K.J., Harfouche M., Abu-Raddad L.J. (2023). Epidemiology of Herpes Simplex Virus Type 2 in Europe: Systematic Review, Meta-Analyses, and Meta-Regressions. Lancet Reg. Health Eur..

[4] Herpes Simplex Virus|WHO. https://www.who.int/news-room/fact-sheets/detail/herpes-simplex-virus.

[5] De Rose D.U., Bompard S., Maddaloni C., Bersani I., Martini L., Santisi A., Longo D., Ronchetti M.P., Dotta A., Auriti C. (2023). Neonatal Herpes Simplex Virus Infection: From the Maternal Infection to the Child Outcome. J. Med. Virol..

[6] Teutsch S., Berkhout A., Raynes-Greenow C., Zurynski Y., Britton P.N., Jones C.A., APSU Neonatal HSV study advisory group (2023). Characteristics of Neonatal Herpes Simplex Central Nervous System Disease in Australia (1997–2020). J. Clin. Virol..

[7] Azeem A., Baartman B., Conrady C.D., Meier J.L., El-Herte R. (2023). Herpes Simplex Virus Dissemination with Necrotizing Hepatitis Following Descemet Membrane Endothelial Keratoplasty. BMC Infect. Dis..

[8] Mazzotta E., Fiorda Diaz J., Echeverria-Villalobos M., Eisinger G., Sprauer S., Singha A., Lyaker M.R. (2022). Case Report: Disseminated Herpes Simplex Virus 1 Infection and Hemophagocytic Lymphohistiocytosis after Immunomodulatory Therapy in a Patient with Coronavirus Disease 2019. Front. Med. (Lausanne).

[9] Kim M., Jalal A., Rubio-Gomez H., Bromberg R. (2022). A Case Report of Severe Systemic Herpes Simplex Virus-1 (HSV-1) Infection with Multi-Organ Involvement after a Course of Oral Corticosteroid Treatment. BMC Infect. Dis..

[10] Silva S., Ayoub H.H., Johnston C., Atun R., Abu-Raddad L.J. (2022). Estimated Economic Burden of Genital Herpes and HIV Attributable to Herpes Simplex Virus Type 2 Infections in 90 Low- and Middle-Income Countries: A Modeling Study. PLoS Med..

[11] Stone J., Looker K.J., Silhol R., Turner K.M.E., Hayes R., Coetzee J., Baral S., Schwartz S., Mayaud P., Gottlieb S. (2023). The Population Impact of Herpes Simplex Virus Type 2 (HSV-2) Vaccination on the Incidence of HSV-2, HIV and Genital Ulcer Disease in South Africa: A Mathematical Modelling Study. EBioMedicine.

[12] Schiffer J.T., Gottlieb S.L. (2019). Biologic Interactions between HSV-2 and HIV-1 and Possible Implications for HSV Vaccine Development. Vaccine.

[13] Looker K.J., Elmes J.A.R., Gottlieb S.L., Schiffer J.T., Vickerman P., Turner K.M.E., Boily M.-C. (2017). Effect of HSV-2 Infection on Subsequent HIV Acquisition: An Updated Systematic Review and Meta-Analysis. Lancet Infect. Dis..

[14] Looker K.J., Welton N.J., Sabin K.M., Dalal S., Vickerman P., Turner K.M.E., Boily M.-C., Gottlieb S.L. (2020). Global and Regional Estimates of the Contribution of Herpes Simplex Virus Type 2 Infection to HIV Incidence: A Population Attributable Fraction Analysis Using Published Epidemiological Data. Lancet Infect. Dis..

[15] Van Wagoner N., Qushair F., Johnston C. (2023). Genital Herpes Infection: Progress and Problems. Infect. Dis. Clin. N. Am..

[16] Herpes—STI Treatment Guidelines. https://www.cdc.gov/std/treatment-guidelines/herpes.htm.

[17] Bras A.P., Sitar D.S., Aoki F.Y. (2001). Comparative Bioavailability of Acyclovir from Oral Valacyclovir and Acyclovir in Patients Treated for Recurrent Genital Herpes Simplex Virus Infection. Can. J. Clin. Pharmacol..

[18] Kumar R., Sinha V.R. (2017). Lipid Nanocarrier: An Efficient Approach Towards Ocular Delivery of Hydrophilic Drug (Valacyclovir). AAPS PharmSciTech.

[19] Osmałek T., Froelich A., Jadach B., Tatarek A., Gadzinski P., Falana A., Gralinska K., Ekert M., Puri V., Wrotynska-Barczynska J. (2021). Recent Advances in Polymer-Based Vaginal Drug Delivery Systems. Pharmaceutics.

[20] Mahant S., Sharma A.K., Gandhi H., Wadhwa R., Dua K., Kapoor D.N. (2022). Emerging Trends and Potential Prospects in Vaginal Drug Delivery. Curr. Drug Deliv..

[21] Xie L., Li Y., Liu Y., Chai Z., Ding Y., Shi L., Wang J. (2023). Vaginal Drug Delivery Systems to Control Microbe-Associated Infections. ACS Appl. Bio Mater..

[22] Pradhan D., Biswasroy P., Goyal A., Ghosh G., Rath G. (2021). Recent Advancement in Nanotechnology-Based Drug Delivery System against Viral Infections. AAPS PharmSciTech.

[23] Chakravarty M., Vora A. (2020). Nanotechnology-Based Antiviral Therapeutics. Drug Deliv. Transl. Res..

[24] Delshadi R., Bahrami A., McClements D.J., Moore M.D., Williams L. (2021). Development of Nanoparticle-Delivery Systems for Antiviral Agents: A Review. J. Control. Release.

[25] Lembo D., Donalisio M., Civra A., Argenziano M., Cavalli R. (2017). Nanomedicine Formulations for the Delivery of Antiviral Drugs: A Promising Solution for the Treatment of Viral Infections. Expert Opin. Drug Deliv..

[26] Cojocaru F.D., Botezat D., Gardikiotis I., Uritu C.M., Dodi G., Trandafir L., Rezus C., Rezus E., Tamba B.I., Mihai C.T. (2020). Nanomaterials Designed for Antiviral Drug Delivery Transport across Biological Barriers. Pharmaceutics.

[27] das Neves J., Nunes R., Machado A., Sarmento B. (2015). Polymer-Based Nanocarriers for Vaginal Drug Delivery. Adv. Drug Deliv. Rev..

[28] Araujo V.H.S., de Souza M.P.C., Carvalho G.C., Duarte J.L., Chorilli M. (2021). Chitosan-Based Systems Aimed at Local Application for Vaginal Infections. Carbohydr. Polym..

[29] Rossi S., Vigani B., Sandri G., Bonferoni M.C., Caramella C.M., Ferrari F. (2019). Recent advances in the mucus-interacting approach for vaginal drug delivery: From mucoadhesive to mucus-penetrating nanoparticles. Expert Opin. Drug Deliv..

[30] Boroumand H., Badie F., Mazaheri S., Seyedi Z.S., Nahand J.S., Nejati M., Baghi H.B., Abbasi-Kolli M., Badehnoosh B., Ghandali M. (2021). Chitosan-Based Nanoparticles Against Viral Infections. Front. Cell. Infect. Microbiol..

[31] Hemmingsen L.M., Škalko-Basnet N., Jøraholmen M.W. (2021). The Expanded Role of Chitosan in Localized Antimicrobial Therapy. Mar. Drugs.

[32] Desai N., Rana D., Salave S., Gupta R., Patel P., Karunakaran B., Sharma A., Giri J., Benival D., Kommineni N. (2023). Chitosan: A Potential Biopolymer in Drug Delivery and Biomedical Applications. Pharmaceutics.

[33] Nayak R., Kar B., Ghosh G., Rath G. (2021). Current trends in chitosan based nanopharmaceuticals for topical vaginal therapies. Int. J. Biol. Macromol..

[34] Cavalli R., Soster M., Argenziano M. (2016). Nanobubbles: A Promising Efficient Tool for Therapeutic Delivery. Ther. Deliv..

[35] Shende P., Jain S. (2019). Polymeric Nanodroplets: An Emerging Trend in Gaseous Delivery System. J. Drug Target..

[36] Hansen H.H.W.B., Cha H., Ouyang L., Zhang J., Jin B., Stratton H., Nguyen N.T., An H. (2023). Nanobubble Technologies: Applications in Therapy from Molecular to Cellular Level. Biotechnol. Adv..

[37] Liu X., Shi D., Guo L., Zhou X., Shang M., Sun X., Meng D., Zhao Y., Li J. (2021). Echogenic, Ultrasound-Sensitive Chitosan Nanodroplets for Spatiotemporally Controlled DKK-2 Gene Delivery to Prostate Cancer Cells. Int. J. Nanomed..

[38] Donalisio M., Argenziano M., Rittà M., Bastiancich C., Civra A., Lembo D., Cavalli R. (2020). Acyclovir-Loaded Sulfobutyl Ether-β-Cyclodextrin Decorated Chitosan Nanodroplets for the Local Treatment of HSV-2 Infections. Int. J. Pharm..

[39] Mandras N., Luganini A., Argenziano M., Roana J., Giribaldi G., Tullio V., Cavallo L., Prato M., Cavalli R., Cuffini A.M. (2023). Design, Characterization, and Biological Activities of Erythromycin-Loaded Nanodroplets to Counteract Infected Chronic Wounds Due to Streptococcus Pyogenes. Int. J. Mol. Sci..

[40] Argenziano M., Bressan B., Luganini A., Finesso N., Genova T., Troia A., Giribaldi G., Banche G., Mandras N., Cuffini A.M. (2021). Comparative Evaluation of Different Chitosan Species and Derivatives as Candidate Biomaterials for Oxygen-Loaded Nanodroplet Formulations to Treat Chronic Wounds. Mar. Drugs.

[41] Owen D.H., Katz D.F. (1999). A Vaginal Fluid Simulant. Contraception.

[42] Falavigna M., Pattacini M., Wibel R., Sonvico F., Škalko-Basnet N., Flaten G.E. (2020). The Vaginal-PVPA: A Vaginal Mucosa-Mimicking In Vitro Permeation Tool for Evaluation of Mucoadhesive Formulations. Pharmaceutics.

[43] Toujani M.M., Rittà M., Civra A., Genovese S., Epifano F., Ghram A., Lembo D., Donalisio M. (2018). Inhibition of HSV-2 Infection by Pure Compounds from Thymus Capitatus Extract in Vitro. Phytother. Res..

[44] Cagno V., Sgorbini B., Sanna C., Cagliero C., Ballero M., Civra A., Donalisio M., Bicchi C., Lembo D., Rubiolo P. (2017). In Vitro Anti-Herpes Simplex Virus-2 Activity of Salvia Desoleana Atzei & V. Picci Essential Oil. PLoS ONE.

[45] Cagno V., Donalisio M., Civra A., Cagliero C., Rubiolo P., Lembo D. (2015). In Vitro Evaluation of the Antiviral Properties of Shilajit and Investigation of Its Mechanisms of Action. J. Ethnopharmacol..

[46] Sureram S., Arduino I., Ueoka R., Rittà M., Francese R., Srivibool R., Darshana D., Piel J., Ruchirawat S., Muratori L. (2022). The Peptide A-3302-B Isolated from a Marine Bacterium Micromonospora Sp. Inhibits HSV-2 Infection by Preventing the Viral Egress from Host Cells. Int. J. Mol. Sci..

[47] Marano F., Argenziano M., Frairia R., Adamini A., Bosco O., Rinella L., Fortunati N., Cavalli R., Catalano M.G. (2016). Doxorubicin-Loaded Nanobubbles Combined with Extracorporeal Shock Waves: Basis for a New Drug Delivery Tool in Anaplastic Thyroid Cancer. Thyroid.

[48] Fülöp Z., Saokham P., Loftsson T. (2014). Sulfobutylether-β-Cyclodextrin/Chitosan Nano- and Microparticles and Their Physicochemical Characteristics. Int. J. Pharm..

[49] Ricci F., Racaniello G.F., Lopedota A., Laquintana V., Arduino I., Lopalco A., Cutrignelli A., Franco M., Sigurdsson H.H., Denora N. (2022). Chitosan/Sulfobutylether-β-Cyclodextrin Based Nanoparticles Coated with Thiolated Hyaluronic Acid for Indomethacin Ophthalmic Delivery. Int. J. Pharm..

[50] Mikušová V., Mikuš P. (2021). Advances in Chitosan-Based Nanoparticles for Drug Delivery. Int. J. Mol. Sci..

[51] De Gaetano F., d’Avanzo N., Mancuso A., De Gaetano A., Paladini G., Caridi F., Venuti V., Paolino D., Ventura C.A. (2023). Chitosan/Cyclodextrin Nanospheres for Potential Nose-to-Brain Targeting of Idebenone. Pharmaceuticals.

[52] Sigurdsson H.H., Knudsen E., Loftsson T., Leeves N., Sigurjonsdottir J.F., Másson M. (2002). Mucoadhesive Sustained Drug Delivery System Based on Cationic Polymer and Anionic Cyclodextrin/Triclosan Complex. J. Incl. Phenom..

[53] Mura P., Maestrelli F., Cirri M., Mennini N. (2022). Multiple Roles of Chitosan in Mucosal Drug Delivery: An Updated Review. Mar. Drugs.

[54] Cazorla-Luna R., Martín-Illana A., Notario-Pérez F., Ruiz-Caro R., Veiga M.D. (2021). Naturally Occurring Polyelectrolytes and Their Use for the Development of Complex-Based Mucoadhesive Drug Delivery Systems: An Overview. Polymers.

[55] Kumar A., Naik P.K., Pradhan D., Ghosh G., Rath G. (2020). Mucoadhesive Formulations: Innovations, Merits, Drawbacks, and Future Outlook. Pharm. Dev. Technol..

[56] Valamla B., Thakor P., Phuse R., Dalvi M., Kharat P., Kumar A., Mehra N.K. (2022). Engineering drug delivery systems to overcome the vaginal mucosal barrier: Current understanding and research agenda of mucoadhesive formulations of vaginal delivery. J. Drug Deliv. Sci. Technol..

[57] Baroni S., Argenziano M., La Cava F., Soster M., Garello F., Lembo D., Cavalli R., Terreno E. (2023). Hard-Shelled Glycol Chitosan Nanoparticles for Dual MRI/US Detection of Drug Delivery/Release: A Proof-of-Concept Study. Nanomaterials.

[58] Chemaly R.F., Hill J.A., Voigt S., Peggs K.S. (2019). In Vitro Comparison of Currently Available and Investigational Antiviral Agents against Pathogenic Human Double-Stranded DNA Viruses: A Systematic Literature Review. Antivir. Res..

[59] Otake T., Schols D., Witvrouw M., Naesens L., Nakashima H., Moriya T., Kurita H., Matsumoto K., Ueba N., De Clercq E. (1994). Modified Cyclodextrin Sulphates(MCDS11) Have Potent Inhibitory Activity against HIV and High Oral Bioavailability. Antivir. Chem. Chemother..

[60] Goncharova E.P., Kostyro Y.A., Ivanov A.V., Zenkova M.A. (2019). A Novel Sulfonated Derivative of β-Cyclodextrin Effectively Inhibits Influenza A Virus Infection in Vitro and in Vivo. Acta Naturae.

[61] Gao J., Yan X., Banfield B.W. (2018). Comparative Analysis of UL16 Mutants Derived from Multiple Strains of Herpes Simplex Virus 2 (HSV-2) and HSV-1 Reveals Species-Specific Requirements for the UL16 Protein. J. Virol..

[62] Leary J.J., Wittrock R., Sarisky R.T., Weinberg A., Levin M.J. (2002). Susceptibilities of Herpes Simplex Viruses to Penciclovir and Acyclovir in Eight Cell Lines. Antimicrob. Agents Chemother..

[63] Jones S.T., Cagno V., Janeček M., Ortiz D., Gasilova N., Piret J., Gasbarri M., Constant D.A., Han Y., Vuković L. (2020). Modified Cyclodextrins as Broad-Spectrum Antivirals. Sci. Adv..

