# Supplementary material for: Enhanced Anti-Herpetic Activity of Valacyclovir Loaded in Sulfobutyl-ether-β-cyclodextrin-decorated Chitosan Nanodroplets
Source: Microorganisms. 2023 Sep 30;11(10):2460. doi: 10.3390/microorganisms11102460 (PMC10609596; doi:10.3390/microorganisms11102460)
Supplement: Supplementary file 1 [file microorganisms-11-02460-s001.zip › microorganisms-2637321-supplementary.pdf]

Table S1. Viral titers of virus yield reduction assays, expressed as Log10(PFU/mL) ± standard deviation (SD).

Formulation	Untreated	14.8 μM	3.7 μM	2.3 μM	0.9 μM
VACV	5.75 ± 0.004	2.78 ± 0.3	3.01 ± 0.5	3.90 ± 0.1	4.98 ± 0.3
SBEβCD-ND-VACV	6.07 ± 0.06	2.78 ± 0.05	2.83 ± 0.07	3.62 ± 0.4	4.39 ± 0.01
SBEβCD-ND	6.08 ± 0.1	5.32 ± 0.07	6.17 ± 0.1	6.12 ± 0.1	5.78 ± 0.1
